# Belief Revision and Delusions: How Do Patients with Schizophrenia Take Advice?

**DOI:** 10.1371/journal.pone.0034771

**Published:** 2012-04-20

**Authors:** Mariia Kaliuzhna, Valérian Chambon, Nicolas Franck, Bérangère Testud, Jean-Baptiste Van der Henst

**Affiliations:** 1 Centre National de la Recherche Scientifique, Laboratoire Langage, Cerveau et Cognition (L2C2), Université de Lyon, Bron, France; 2 Laboratory of Cognitive Neuroscience, Brain Mind Institute, Ecole Polytechnique Fédérale de Lausanne, Lausanne, Switzerland; 3 Centre National de la Recherche Scientifique, Centre de Neuroscience Cognitive, Université de Lyon, Bron, France; 4 Centre Hospitalier Le Vinatier, Bron, France; 5 Université Lumière Lyon 2, Bron, France; Catholic University of Sacred Heart of Rome, Italy

## Abstract

The dominant cognitive model that accounts for the persistence of delusional beliefs in schizophrenia postulates that patients suffer from a general deficit in belief revision. It is generally assumed that this deficit is a consequence of impaired reasoning skills. However, the possibility that such inflexibility affects the entire system of a patient's beliefs has rarely been empirically tested. Using delusion-neutral material in a well-documented advice-taking task, the present study reports that patients with schizophrenia: 1) revise their beliefs, 2) take into account socially provided information to do so, 3) are not overconfident about their judgments, and 4) show less egocentric advice-discounting than controls. This study thus shows that delusional patients' difficulty in revising beliefs is more selective than had been previously assumed. The specificities of the task and the implications for a theory of delusion formation are discussed.

## Introduction

Belief change is a complex process by which rational agents shift from one belief state to another as a way to improve their knowledge [1]. Every day we deal with a lot of information coming from different sources that may have an impact on our belief system. One obvious way belief change occurs is the expansion of our belief set when we acquire new information. However, quite often a new piece of information contradicts our knowledge and in order to preserve the consistency of our beliefs we must be able to consider that some of them are untrue and ought to be abandoned. Evaluating and revising beliefs is a crucial ability as it plays a central role in the flexibility of human cognition.

One can reasonably assume that a dysfunction in such a mechanism would prevent an individual from correctly assessing her/his beliefs and eliminating those that are patently false in light of compelling counter-evidence. This could lead her/him to hold ill-founded, irrational or even *delusional beliefs*. The latter are defined as rationally untenable beliefs based on incorrect inference about reality. These beliefs persist despite the evidence to the contrary and are not ordinarily accepted by other members of the person's culture or subculture [2], [3]. Deficit in belief revision will not cause the abnormal or unrealistic content of these beliefs by itself, but it could make these beliefs hard to assess and reject. This idea has been largely developed by Langdon and Coltheart [4] (see also [5], [6]). They argue that the process of experiencing a delusion is a composite of several impairments that cannot independently explain the presence of a delusional belief. On the one hand, they admit that perceptual aberrations, potentially coupled with attributional biases, strongly contribute to generate the content of bizarre delusions. On the other hand, they emphasize that such factors are not sufficient to explain why delusional beliefs are deeply entrenched instead of being ephemeral hypotheses doomed to disappear. In particular they argue that having an anomalous experience should not prevent individuals from accepting the possibility that some dysfunction arises in their mind and that their experience is thus inaccurate. Consistent with this view they provide numerous examples of brain dysfunctions that result in anomalous experiences without resulting in delusional beliefs. For instance amputated patients who feel the anomalous presence of their missing limb certainly suffer from some neurophysiological dysfunction but do not firmly believe that their limb is still there and do not become delusional. Even more strikingly, some brain-damaged non-deluded patients may go through anomalous experiences that are very similar to those of deluded patients. It is thus not the case that experiencing a serious perceptual anomaly leads to delusion.

To account for delusions, the authors thus postulate the existence of another factor, namely a deficit in belief evaluation and revision.

In line with this view, clinical observations show that deluded patients, such as those suffering from schizophrenia, tend to hold their delusional beliefs with a high degree of conviction and ignore any evidence, arguments, or outside opinion that could lead them to revise their views [3]–[4], [7]–[9]. For instance, Freeman et al. [8] found that when delusional patients (78% of whom suffered from schizophrenia) were asked to account for the experiences they go through and asked to propose a different explanation than the delusional one, the majority was unable to do so (76 out of 100 patients). The remaining few thought of one single alternative (21 out of 100 patients, and only 3 patients proposed two alternatives).

It has been proposed that the failure to revise one's own delusional beliefs and generate alternative beliefs may result from a deficit in inductive reasoning and hypothesis testing, which manifests itself in reasoning biases [4], [9], [10]. For instance, several studies have reported that patients are prone to jump-to-conclusions (JTC) biases. Indeed, when compared to controls, schizophrenic deluded patients hold beliefs with less evidential support and consequentially make more hasty judgements in decision making tasks. They also show a higher level of confidence when expressing their judgements and tend to make their decisions on the basis of information that is immediately present in the environment, thus ignoring previously acquired knowledge [11], [12]. Interestingly, Freeman et al. [8] reported that delusional patients who were unable to consider alternative explanations were also more prone to the JTC bias than those who did find an alternative (see also [10]). Furthermore, patients tend to exhibit dichotomous thinking and intolerance to ambiguity [10], [13], answering in extreme, absolute terms, such as “completely certain”, or “do not agree at all”. They thus have difficulties evaluating their answers and choosing more measured replies. Such a behavioural tendency could prevent patients from being more cautious while formulating an account of their experiences.

Although impaired reasoning skills could well account for a general deficit in belief revision, the studies in which they are reported do not directly show that delusional patients suffer from such a deficit. However, an experimental study that explicitly addressed this issue was that of Moritz and Woodward [14]. Using non delusional material, they aimed to investigate whether schizophrenic patients are less able to revise their false convictions in general, and not only their delusional beliefs. In the task they devised, delusional patients with schizophrenia saw a sequence of picture fragments that gradually developed into a complete identifiable picture (i.e. at each stage a new fragment appeared so that the picture became increasingly recognisable). After each new fragment, participants were presented with several interpretations, which could match or mismatch the target picture, and had to evaluate their plausibility. For example, a picture progressively showing a mermaid could also initially suggest a fish or a seal. The likelihood of these false interpretations was supposed to decrease over time, whereas the interpretation “person in winter clothing” was highly improbable at any stage. The results indicated that schizophrenic patients did not diminish their probability ratings regarding the interpretations that became implausible (e.g. the fish) to the same extent as did healthy controls and other psychiatric patients. The authors therefore concluded that schizophrenic patients exhibit a general bias against *disconfirmatory* evidence (BADE, see also [15]–[18]).

Although Moritz and Woodward's study [14] is highly valuable, one should refrain from concluding that their results allow one to firmly establish that schizophrenic patients suffer from a *general* deficit in belief revision. First, in their task, patients did show evidence of belief change in the right direction. Indeed, their degree of belief regarding the correct interpretation (i.e. the mermaid) significantly increased over time and this increase was not less pronounced than for control participants. In other words, they did not show any bias against *confirmatory* evidence (BACE) and the authors did not report any evidence that their patients discovered the correct interpretation with less ease than their control participants. This result is a marker of a revision process since patients show a preference for one answer over others, and this preference is supported by a new piece of evidence disclosed as the picture becomes identifiable.

Second, other cognitive deficits than a belief revision impairment might account for the BADE score. It is well-established that schizophrenic patients have difficulties in focussing their attention and in overcoming potentially disruptive stimuli [19], [20]. In order to obtain a high BADE score, participants in Moritz & Woodward's task [14] need to pay attention to the entire set of proposed interpretations. Given the relatively high number of interpretations provided (up to 9), it might thus be the case that patients focus more on the correct interpretation and subsequently pay less attention to their evaluations of the incorrect interpretations.

Third, in order to establish a general deficit one needs to explore various situations where such a deficit is likely to arise. Since each task has its own peculiarities, any deficit reported in a given situation may be specific to that situation. The greater the variety of situations showing the same kind of deficit, the greater the chance that such a deficit is general. As indicated below, it might be the case that the belief revision impairment only afflicts a selected category of beliefs. For reasons we suggest in the discussion, this particular set of beliefs will be more difficult to revise and will thus constitute what is called delusions. The work of Woodward and Moritz is thus of high relevance since it explores belief revision when more neutral beliefs are formed. However, their task obviously involves its own specific characteristics and thus one needs to go one step further and explore situations including different features.

In the present study, beliefs were related to encyclopaedic knowledge and the context of revision was that of an *advice taking* situation. Since delusional beliefs are usually not accepted by other members of the patients' culture or subculture [2], patients often come across other people's opinions (family members, friends, clinicians, etc.) which defy their own beliefs. Patients seem impervious to arguments and suggestions provided by others, and strongly reject objections to their delusions [9]. For instance, clinical investigations of patients' insight regarding their anomalous experiences [21] , indicate that deluded schizophrenic patients score low on items that test their receptiveness to correction from others (some of these items were: (9) I know better than anyone else what my problems are. (10) When people disagree with me, they are generally wrong. (11) I cannot trust other people's opinion about my experiences. (12) If somebody points out that my beliefs are wrong, I am willing to consider it) (see also [22]; and see [23] for a review). Patients may even see critics as members of a conspiracy [14].

In the wake of Moritz and Woodward's work [14], the present study aims to investigate whether the tendency to reject others' opinions is *general* and extends to non-delusional beliefs. We thus explore a very basic and well-investigated situation of social influence that may lead people to revise their views: the individual produces an initial estimate (usually quantitative) in a certain task, then receives the estimate of another person, and ultimately makes a final estimate. This typically illustrates what is referred to as an *advice taking* situation in the decision making literature. The difference between the initial and the final estimates offers a measure of the extent to which people combine advice with their own opinion. Results obtained with healthy participants indicate that the weight assigned to advice varies according to a number of factors such as the trustworthiness of the adviser [24] her/his expertise [25], previous suggestions made by the adviser (i.e. her/his reputation [26]), the distance of the advice from the others' opinions [27], the number of advisers [28], etc. (see [29], for a review). However, the main result in this field is that participants show an *egocentric advice discounting* (EAD, as phrased by Yaniv) as they tend to outweight their own opinion as compared to that of the advisor(s). For example, if participant's initial opinions are assigned the value of 0, and the advice the value of 100, the final opinions tend to be situated around 30 on average [26], [27], [30], [31].

The present experiment involves a task devised by Yaniv [26], [27] which consists of responding to general knowledge questions concerning dates of historical and popular events such as, “When did Marilyn Monroe die?”. After completing the questionnaire, participants are once more presented with the same questions but this time they also know other participants' answers. According to the general belief revision deficit hypothesis, deluded schizophrenic patients should be less likely to take advice into account and should display a stronger EAD than healthy controls. Alternatively, this should not be necessarily the case if such a deficit is not general and only affects a particular category of beliefs Along with testing these hypotheses, the present experiment also aims to investigate how the assimilation and rejection of advice relates to the severity of the symptoms.

## Methods

### Ethics Statement

All participants gave written informed consent for the study which was approved by the local Ethical Committee (Comité de Protection des Personnes, no. AFSSAPS: 2008-A01599-46). All patients consented to participate in the research on their own behalf. Prior to the task, the experimenters made sure that patients had the capacity to consent by asking a series of questions about their understanding of the study.

### Participants

Thirty patients with schizophrenia (22 males, 8 females; mean age: 37.3 years, SD: 8.8) and 30 control participants (17 males, 13 females, mean age: 39.6 years, SD: 11.7) participated in the study. All patients fulfilled DSM-IV criteria of schizophrenia [2] with no other psychiatric diagnosis on DSM-IV Axis I. Exclusion criteria included history of neurological illness or trauma, alcohol or drug dependence according to DSM-IV criteria, analphabetism, and being over 60 years of age. All patients were receiving antipsychotic medication and were clinically stable at the time of testing (duration of illness: mean: 8 years, SD: 5.12). Comparison participants reported no psychiatric problems, and were systematically matched with patients for sex, age, and years of education (see Table 1). None of the participants were paid for taking part in the study.

**Table 1:** Clinical and demographic characteristics of patients and controls.

	Healthy controls N = 30	Patients N = 30	*P* value
Mean age in years (*SD*)	39.6 (11.7)	37.3 (8.8)	0.4
Mean years of education (*SD*)	12 (1.91)	11.6 (2.17)	0.45
Sex ratio (M/F)	17/13	22/8	0.18
Duration of illness in years *(SD)*		8 (5.1)	
SAPS score *(SD)*		29 (27.4)	
SANS score *(SD)*		31 (17.9)	
Hallucinations score *(SD)*		4.5 (6.5)	
Delusion score *(SD)*		11.9 (12.9)	
Bizarre Behavior score *(SD)*		3.6 (3.2)	
Formal thought disorder score *(SD)*		7.3 (8.8)	
Affective flattening score *(SD)*		9 (7.9)	
Alogia score *(SD)*		4.9 (5.4)	
Avolition score *(SD)*		4.4 (3)	
Ahnedonia-asociality score *(SD)*		8 (4.9)	
Attention		1.5 (1.7)	

### Clinical assessment

SAPS [32] and SANS [33] were used to obtain ratings for positive and negative symptoms in the schizophrenia sample (the mean scores are presented in Table 1). A disorganization score was also computed by summing the following subscores: bizarre behaviour, positive formal thought disorder (from the SAPS), alogia and inappropriate affect (from the SANS). These items have been shown to constitute regular and fundamental components of the disorganization dimension [34]. Two patients had a SAPS delusion subscore of 0 (no delusions). For the remaining patients, the delusion subscore ranged from 2 (mild delusion) to 43 (severe delusion).

### Procedure and materials

**Figure 1:**
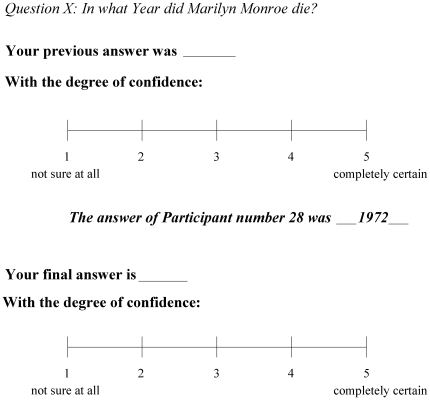
Example of the second part of the experimental procedure. Participants had to copy their initial answer and degree of confidence, read the advice presented and then give their final estimate along with a final confidence rating.

Following Yaniv [26], [27] we conceived a set of 35 general knowledge questions related to world history (e.g. When was UNO created?), French history (When was Napoleon Bonaparte crowned emperor?) as well as famous popular cultural events (When did Marilyn Monroe die? See Appendix S1, for the full set of questions). The 35 questions were presented in French to control participants and patients. In the first phase, participants read these questions in a booklet and had to answer them by indicating the year in which they thought each historical event took place. Additionally, they also had to estimate their degree of confidence in their reply on a 5 point Likert scale (1 – not sure at all; 5 – completely certain). “Once the questionnaire was completed the second phase began. Participants were told that they would be given the same questions once again (in a new booklet) but this time every question would be accompanied by an estimate provided by another individual (i.e. the advice). Their task was now to: 1) copy their initial answer and their initial degree of confidence (i.e. those given in the first questionnaire) into the new booklet, 2) read the new estimate presented (i.e. the advice) and 3) give their final estimate that could differ, or not, from their first one, accompanied by the degree of confidence in the new answer.” (for an example of a trial in the second phase, see Figure 1).

We did not indicate to participants whether the advice was obtained from patients or from healthy individuals. They were only told that those estimates came from people who were of approximately the same age and education level as themselves and who also took part in this study. We considered that providing more details about the population of advisers might have biased the extent to which they would have taken the advice into account. On one hand, had we indicated that the opinions were obtained from healthy participants, patients may have considered these advisers to be more knowledgeable than them. On the other hand, had we presented the advisers as suffering from any sort of disease, patients might have considered these advisers as less competent. It turned out that during the experiment, neither patients nor controls inquired about who gave the advice. Following Yaniv ([27], Experiments 1 and 2) the 35 advice estimates were actual responses provided by other individuals and they were presented as being obtained from different individuals, each labelled by a different number. Accuracy of the advice, which was measured by the absolute number of years away from the correct response, was 12.3 years. During the task, participants were not given feedback regarding their own level of accuracy nor did they receive any information about the adviser's degree of accuracy. It is only in the debriefing phase that the correct dates could be shown to participants.

## Results

### Accuracy and confidence: patients and controls perform in a similar way

**Figure 2:**
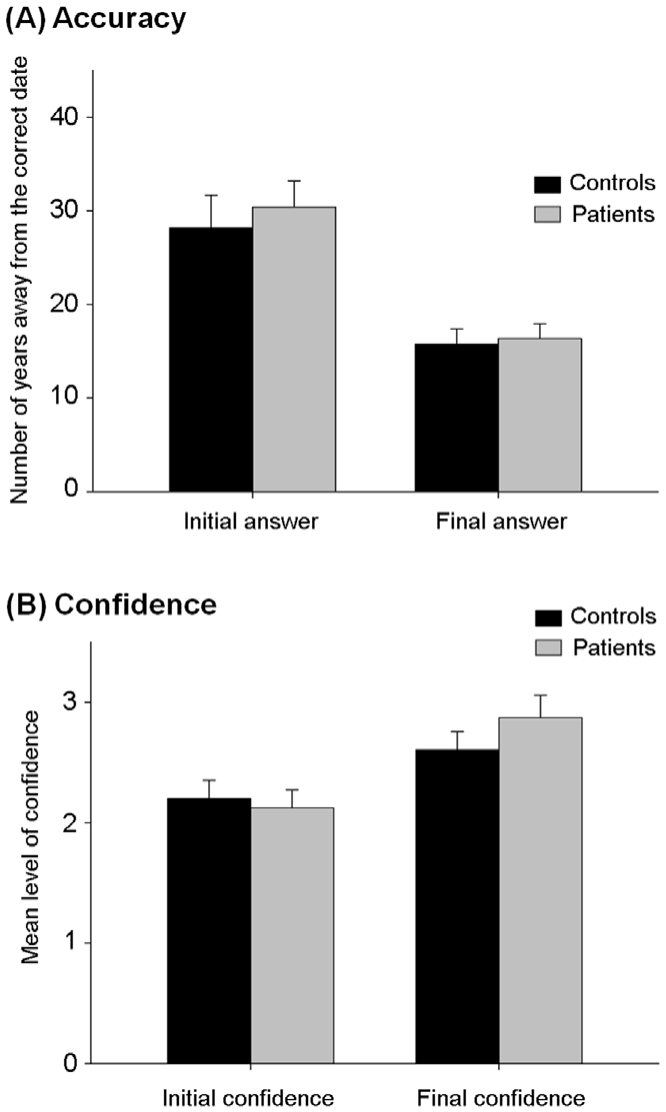
Accuracy and confidence: patients and controls perform in a similar way. Accuracy (**A**): Mean number of years away from the correct date; Confidence (**B**): Mean level of confidence. Bars indicate standard error means.

Accuracy was assessed by the measure of the absolute number of years away from the correct response. For each participant the averaged accuracy across the 35 questions was calculated. In the control group the averaged accuracy was 28.2 years for the initial answer and 15.8 years for the final answer. In the group of patients, the averaged accuracy was 30.4 years for the initial answer and 16.4 years for the final answer. Hence, participants of the two groups showed a similar level of performance and tended to change their answer and improved their performance after receiving the advice (see Figure 2A). This was confirmed by running an ANOVA with accuracy as the dependant variable, participants' group (Controls vs. Patients) as the between-subject factor and the time of the question (before vs. after the advice) as a within-subject factor. The ANOVA shows a main effect of the time of the question (F (1,58) = 45.21, p<10^−5^) but no main effect of group (F (1,58) = 0.23, p>0.6) nor significant interaction between these two factors (F (1,58) = 0.18, p>0.6). The improvement of performance can easily be explained by the fact that the advice estimates were much more accurate than the initial responses of the participants for both groups: 16.9 years better for the control group (t-test = 4.69, p<10-4) and 17.1 years for the group of patients (17.1 years better, t-test = 5.61, p<10-6). Note that the difference of performance between controls and their advice was similar to the difference of performance between patients and their advice (16.9 years vs. 17.1 years, t-test (58) = 0.04, p>0.9). The absence of difference in performance in initial answers was not only quantitative but it was also qualitative as the difficulty orders of the questions highly correlated between the two groups (r = 0.88, p<10^−5^) This means that both groups had lower and higher accuracy for the same questions: difficult questions (i.e. those which had the lowest level of accuracy) for the patients were also difficult for the controls. Finally, the level of accuracy (as measured by the number of years away from the correct answer) and the number of education years did not significantly correlate and for patients, the level of accuracy did not significantly correlate with any of the clinical scores (all *R's*>−.32 and <.17, all p's>.07).

The level of confidence was also similar across the two groups and both controls and patients increased their confidence after receiving the advice (see Figure 2B): The level of confidence rose from 2.20 to 2.60 among controls and from to 2.12 to 2.87 among patients. This pattern was confirmed by running an ANOVA with the level of confidence as the dependent variable, the participants' group (Controls vs. Patients) as a between-subject factor and the time of the question (before vs. after the advice) as a within-subject factor. The ANOVA shows a main effect of the time of the question (F (1,58) = 31.9, p<10^−4^) but no main effect of group (F (1,58) = 0.23, p>0.6) and a marginally significant interaction between these two factors (F (1,58) = 2.86, p = 0.096) indicating that controls' confidence increased a bit more than that of patients.

### Weight of advice: patients put weight more on the advice than control participants

The weight of advice (WOA) is our main variable of interest as it is a measure of the extent to which participants took the advice into account in their final estimate. It is calculated with the following formula [27]:
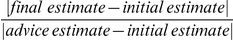
The WOA ranges from 0 to 1: it is equal to 0 when the participant totally ignores the advice and proposes a final answer that is identical to her/his initial answer; it is equal to 1 when the participant gives up her/his initial answer and completely follows the estimate of the advisor. It is equal to 0.5 when equal weights are assigned to each opinion.

**Figure 3:**
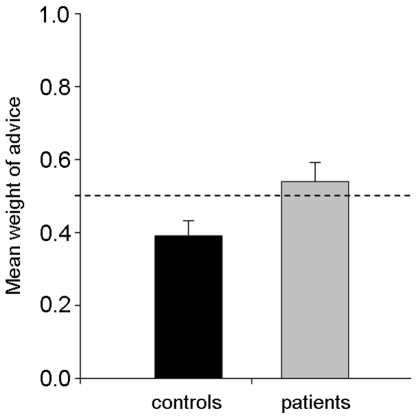
Patients put weight more on the advice than control participants. The dashed line represents equal weight ascribed to advice and initial opinions. Bars indicate standard error means.

In order to obtain a well-defined estimate of WOA, the following two criteria have to be met. First, the advice should differ from the initial answer; otherwise one cannot distinguish between a final answer sticking to the initial response and a final answer adhering to the advice. Because the advice estimates were randomly taken from a pool of actual estimates that were selected before knowing participants' answers, we were exposed to the risk of such equalities. Indeed, 2.33% of the answers (2.29% of answers in the Control group and 2.38% in the group of patients) turned out to be identical to the advice and were thus excluded from the WOA analysis. Second, the final answer should fall between the initial answer and the advice. Exceptions to such criterion include 1) final answers which are even further away from the advice than the initial answer (e.g. the participant initially answered 1970, s/he received 1975 as an advice, and then finally answered 1968) and 2) final answers which are even further away from the initial answer than the advice (e.g. the participant initially answered 1970, s/he received 1975 as an advice, and then finally answered 1977). The rate of answers that did not meet this criterion represented 6.29% of the total answers, (i.e. 5.62% of answers in the Control group and 6.95% in the group of patients, t-test on the arcsin-transformed proportions, t (58) = 0.64, p>0.5) which were excluded from the WOA analysis. Once these two exclusion criteria were applied, we calculated, for each participant, the mean WOA across the 35 questions. In line with the observations reported by the literature, participants in the control group showed egocentric advice discounting [27] as they put more weight on their own estimate than on the advisor's estimate: the mean weight of advice for this group (0.39) was significantly lower than 0.5 (t test on the arcsin-transformed proportions, t test(29) = −2.71, p<0.02 two-tailed). In contrast, patients showed a more equal weighting between the two opinions: the mean weight of advice for this group (0.54) did not significantly differ from 0.5 (t test on the arcsin-transformed proportions, t test(29) = 0.7, p>0.4). Moreover, the comparison between the two groups shows that Patients' WOA is higher than Controls' WOA (0.39 vs. 0.54, t test on the arcsin-transformed proportions, t (58) = 2.1, p<0.05, see Figure 3). Such a result obviously does not support the prediction that patients should stick more with their initial beliefs than controls. Also note that for patients the mean WOA and the duration of illness did not significantly correlate (r = 0.08, p>0.6).

The mean weight of advice may leave the impression that participants combine their own estimate to the advisor's estimates. For instance, a mean WOA of 0.30 could indicate that for each trial participants modify their initial answer in such a way that about 30% of the distance towards the advice has been covered. However, as noted by Soll and Larrick [35] the distribution underlying the WOA mean may be completely different. For instance, participants could stick entirely with their own opinion for 70% of the questions and fully adhere to the opinion of the advisor for the remaining 30%. Such a pattern would be inconsistent with the idea that most final estimates result from a combination process but instead would reflect a *choosing strategy*. In fact, fine-grained analyses of the WOA distributions indicated that the level of choosing was quite high and was largely underestimated in past studies (see [35]). We thus looked more carefully at the distribution underlying the WOA by examining the 967 individual data points of the control group ((30 participants×35 questions) – 83 answers that did not meet the two above criteria) and the 952 data points of the group of patients ((30 participants×35 questions) – 98 answers that did not meet the two above criteria). As shown in Table 2, this analysis revealed that the rate of choosing was high for both groups: 80.4% of the answers in the control group followed a choosing strategy (WOA = 0 or 1) and 81.4% of the answers in the group of patients followed a choosing strategy. Moreover, in line with the difference regarding the mean WOA between the two groups, the modal WOA was equal to 0 among the group of controls while it was equal to 1 among the group of patients (see Table 2). This again shows that patients adhered more to the advisor's estimate than controls.

**Table 2:** Proportions of choosing and averaging in the two groups.

	Choosing		Averaging	
	Sticking with one's own opinion (WOA = 0)	Adopting the opinion of the advisor (WOA = 1)	Combining one's own opinion with that of the advisor (0<WOA<1)	Total
Individual data point				
**Controls** *N* = 967	52,84%	27,51%	19,65%	**100%**
Individual data point				
**Patients** *N* = 952	36,45%	44,85%	18,70%	**100%**
**Total**	**44,71%**	**36,11%**	**19,18%**	**100%**

Finally, we analysed the relation between confidence and the mean WOA. First, we examined the relation between the initial level of confidence and the mean WOA. In both groups the two measures were negatively correlated (controls r = −0.48, p<.01; patients r = −0.44, p<.02) indicating that the less confident the participants in their initial estimates, the more likely they are to take advice into account. Second, we examined the relation between the increase of confidence over time and the mean WOA. In both groups the two measures were positively correlated (controls r = 0.52, p = .003; patients r = 0.51, p<.005) indicating that the more participants take advice into account the more their level of confidence increases.

### Regression analyses: The Weight of Advice predicts the severity of clinical symptoms

Regression analyses were conducted to evaluate the influence of WOA on patients' clinical symptoms. In particular, we assessed whether the WOA was predictive of the symptom severity on the different dimensions of schizophrenia measured (SANS, SAPS, and disorganisation scores). For each clinical score, we conducted regression analyses using the mean WOA as a predictor variable. We used both this raw score (simple linear regressions), or its transformed values (simple non-linear regressions with logarithmic, polynomial, or exponential transformations). Models with the highest adjusted R-squared (*R* ^2^) and a *p*-value<0.05 are reported.

**Figure 4:**
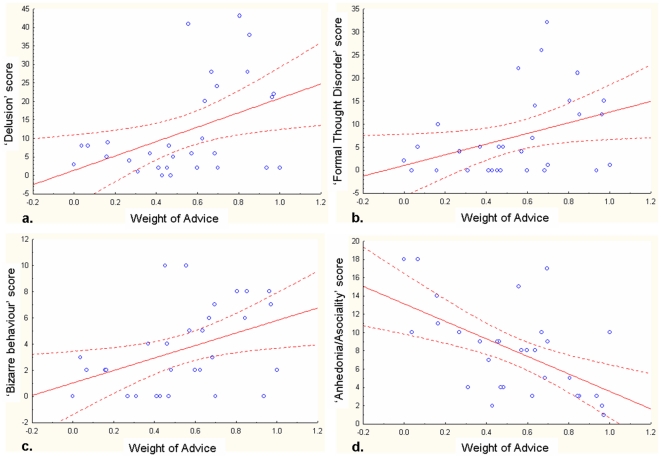
The Weight of Advice predicts the severity of clinical symptoms. The linear regression lines derived from the linear regressions analyses between the Weight of Advice (explanatory factor) and patients’ symptoms are shown in red. The 95% confidence intervals for the regression lines are shown in grey. Note that the correlation is positive for SAPS subscores (**a, b, c**), and negative for the SANS subscore (**d**).

The mean WOA significantly and positively predicted the SAPS score (*R^2^* = 0.17, p = 0.024) and several of its subscores, namely the delusion subscore (*R^2^* = 0.19, p<0.017), the bizarre behaviour subscore (*R^2^* = 0.18, p = 0.02) and the formal thought disorder subscore (*R^2^* = 0.14, p = 0.04, see Figure 4). Thus, the more patients followed the advisor's opinion (and concomitantly gave up her/his opinion), the more severe were those symptoms. The mean WOA significantly and negatively predicted the anhedonia-asociality subscore from the SANS (*R^2^* = −0.32, p<0.002, see Figure 4). The disorganisation score was not found to be predicted by patients' WOA. This was also true when the WOA scores were transformed.

## Discussion

The purpose of this study was to investigate the extent to which delusional patients with schizophrenia modified their beliefs after learning other people's opinions. As in Moritz and Woodward's study [14], the beliefs at stake in the present experiment were not delusion-specific nor were they emotionally charged but involved neutral encyclopaedic knowledge. Participants were confronted with other people's opinions that could lead them to revise their own views. The hypothesis of a general belief revision deficit predicts that patients should stick more to their initial beliefs and should thus reject more advice than control participants. The data indicate that this was not the case as the opposite effect was actually observed. Indeed, only control participants, but not patients, did show evidence of Egocentric Advice Discounting. Both groups changed their initial answer, but patients changed it significantly more than controls. The strong tendency to reject others' opinions, which was previously reported when delusional beliefs are at stake, did not manifest itself with the delusion-neutral material used in this study. These data are thus at odds with the hypothesis of a general belief revision deficit.

It is important to note that the difference between the two groups with respect to WOA cannot be accounted for by a difference in initial performance since both groups did equally well. Had the patients been less accurate than controls in their first answers they might have been also less confident and thus more likely to take advice. One should also note that along with the same level of performance, patients and controls also showed the same degree of confidence in their initial answers. This factor cannot therefore account for the difference in WOA either. Moreover, the level of confidence expressed by the patients turned out to be quite reasonable and mirrored that of controls. In both groups, participants were more likely to take advice into account when their confidence in the initial answer was low, and in both groups confidence rose with the extent to which advice was taken into account. However, patients' confidence increased slightly more than that of the controls. This can easily be explained by a general tendency (observed in both groups) to increase one's own confidence when taking advice more into account. Since patients showed a greater WOA they also ended up with a greater increase of confidence. In short, measures of confidence indicate that patients did consider advice to be worthwhile. Ultimately, the absence of overconfidence in patients' initial judgements also challenges the general deficit view according to which greater belief entrenchment could have been expected in patients. Also, the greater WOA for patients is unlikely to be accounted for by the fact they are immersed in a medical environment where they are used to following advice from medical staff. Indeed the mean WOA and the duration of illness (which is an indirect, though reliable measure, of duration of hospitalization) did not significantly correlate. Moreover, the greater WOA for patients cannot be accounted for by “weakness of will” either as their mean WOA and Avolition clinical score did not significantly correlate.

How can one account for the fact that patients did actually revise their beliefs? The specificities of the task obviously need to be considered here. First, it should be noted that most of our encyclopaedic beliefs are acquired through social transmission, as we obtain them from various social sources such as teachers, newspapers, TV, books or friends. They thus differ from beliefs acquired only by oneself, which arise from direct perception or inference. In this respect, encyclopaedic beliefs radically differ from delusional beliefs and the kind of hypothetical beliefs involved in Moritz and Woodward's task [14]. The former required trusting other people and accepting information they communicate while the latter are entrenched on individual cognitive mechanisms [36]. One could therefore speculate that patients might show a deficit in belief revision but only when individually acquired beliefs are at stake.

Second, it might be the case that when non-delusional neutral beliefs are at stake, being in a situation where advice is available may help patients revise these beliefs. This would again indicate that when hints to revise are embedded within a social context they are more likely to be effective. Such a claim would have to be experimentally tested by comparing the impact of contrary evidence when advocated by an individual to the impact of contrary evidence available in the physical environment as in Moritz & Woodward [14]. Of course, when it comes to delusional beliefs, the social context probably has no impact as patients do not change these beliefs when they are challenged by others [9].

Third, it is important to note that the degree of confidence in the initial answer was relatively low in both groups, and one could therefore conjecture that a deficit in belief revision arises only after a certain threshold of belief entrenchment has been exceeded. In line with this possibility, Woodward and colleagues [18] reported that schizophrenia patients were actually willing to revise their false beliefs when those beliefs were weakly entrenched and observed that the bias against disconfirmatory evidence only occurred with strongly held beliefs (note that such a finding challenges a strong version of the *general* deficit assumption). Although the present experiment did not aim to manipulate belief strength, the degree of confidence can however be exploited to explore the possibility that a deficit in belief revision concerns only strongly held beliefs. We thus analyzed the WOA when participants were highly confident in their initial answer (i.e. degree of confidence equal or higher than 4 on the 5-point scale). In order to obtain a relatively reliable score for each participant, we only considered patients and controls that reported at least three confident answers, resulting in 17 participants in the control group and 15 in the patient group. Unsurprisingly, both groups discounted advice, but as previously observed, patients did not do so more than control participants (controls: mean WOA = .09; median WOA = 0; patients: mean WOA = .24; median.14; Mann-Whitney U test, p = .14). The data thus do not support the hypothesis that patients would revise their strongly held beliefs less than controls. However, a better controlled experiment could be devised in order to investigate the impact of belief strength in a more systematic way. After all encyclopaedic beliefs are likely to be of a little importance to the participants as they are not personally relevant to them and do not carry rich emotional significance. Hence, even though patients are confident in these beliefs, the willingness to stick with them might be relatively weak. Future research should thus investigate how belief content may differentially affect belief revision in control participants and schizophrenic patients. Overall, a deficit in belief revision may well arise for non-delusional beliefs but the conditions and the type of beliefs that would render such a deficit observable still need to be thoroughly explored.

Finally, an important result that needs to be discussed is the greater WOA for patients. How can one account for such a finding? First of all, it is important to note that not all patients took more advice into account. Regression analyses indicate that patients who were more likely to take advice into account showed greater positive symptoms (especially those who scored high on delusion, bizarre behavior and formal thought disorder) while those who were less likely to take advice scored high on the anhedonia-asociality subscale. One explanation that could account for the greater WOA among patients relates to the way they weight and attend to information accessible in their environment. Past studies indicate that deluded schizophrenic patients tend to weight immediately available information much more than background and contextual knowledge as compared to controls. They are thus less likely to link a stimulus they are currently processing to its proper context. This phenomenon has been referred to as the Immediacy mechanism [37] and has been claimed to account for a number of effects observed among schizophrenic patients (see [38]). For instance, manifestations of such a mechanism occur in language processing, with patients exhibiting a clear penchant for processing words in isolation [39], [40].

In the present task, if patients pay more attention to immediate than to remote stimuli they may therefore ascribe a greater weight to advice than do controls, as advice is the last information available in the environment when patients make their final estimates. According to this account, belief revision is likely to arise when contradictory evidence is the most immediately available piece of information in the patient's environment. In this respect, an earlier study by Garety et al. [12] has reported a similar effect to the one observed here. Indeed, the authors used a well-investigated probabilistic inference task where participants are requested to estimate from which of several jars a sequence of colored beads has been drawn. They observed that schizophrenic as well as paranoid patients were more likely to revise their estimates when they came across an item that disconfirms their initial hypothesis than control participants. For instance, if the first three beads supported the hypothesis of a draw from Jar-A while the fourth bead did not, deluded patients tended to abandon the Jar-A hypothesis more than controls.

In a more recent paper Speechley et al. [41] provide further details for the JTC bias in delusions. The authors show that delusional patients manifest hypersalience of evidence-hypothesis matches, whereas immediate information corroborating a given hypothesis is overaccomodated (gains an abnormal degree of importance). In our task the advice was the last information available before the final answer and could thus be attributed a heightened amount of salience. In the same vein McKay [42] interprets the second factor in delusion formation of Davies et al. [5] theory as a bias toward explanatory adequacy, meaning that the individuals' beliefs are primarily based on the available perceptual information as if independently of prior beliefs. Both of these views would suggest that advice being the last available information in the environment it should have a stronger influence of the patients' responses in comparison to the control group.

In the present task, the very possibility of the Immediacy mechanism will need to be further investigated as the initial opinion might still have been part of the immediate environment. Indeed, the advice and the initial opinion were presented almost simultaneously as patients had to report their initial answer on a line above the advice (a procedure that may involve effort and attention to the initial answer). Moreover, given that there were only two pieces of information (i.e. the initial opinion and the advice) it is quite likely that both could be easily compared within the same attentional window. Finally, even though the Immediacy mechanism could account for belief revision in the present experiment it would still need to be understood why the self (symbolized by the initial opinion) could be so easily isolated from the immediate environment (symbolized by the advice). Indeed, it is quite intuitive to consider that the self is almost always immediately accessible [43]. This leads us to consider another, and possibly complementary, explanation.

Clinical observations and phenomenological accounts report blurred delimitation between self and other in schizophrenia as well as disturbed perception of «myness» (for instance see [44], Case 12). This self-monitoring difficulty is thought to underlie misattribution failure in thought and action (Schneiderian first-rank symptoms, [45]) and serves as a basis for the hypothesis that misattribution of internal speech is the cause of verbal hallucinations [46]. Experimental manipulations with schizophrenic patients show aberrant source monitoring for action representation [47] and recalling of produced vs. heard speech [48], [49]. This difficulty in internally distinguishing between self-generated and externally provided information, along with the Immediacy mechanism, could make patients mistake the advice for part of their own knowledge. That is, when presented with the second questionnaire, the response of some other participant would gain as much salience as the patient's own knowledge, thus making it difficult to actually favor one perspective over the other. More generally, this raises the question of how permeable the thoughts of deluded schizophrenic patients are to others and the extent to which these patients are prone to credulity. In future research it may be worth investigating whether patients lack epistemic vigilance [50] and are vulnerable to deception.

## Supporting Information

Appendix S1 **Full set of general knowledge questions.**
(DOC)
