# Supplementary material for: Belief Revision and Delusions: How Do Patients with Schizophrenia Take Advice?
Source: PLoS One. 2012 Apr 20;7(4):e34771. doi: 10.1371/journal.pone.0034771 (PMC3335042; doi:10.1371/journal.pone.0034771)
Supplement: Appendix S1 — Full set of general knowledge questions. (DOC) [file pone.0034771.s001.doc]

Appendix : The 35 questions

1. In what year was emperor Napoleon Bonaparte crowned?
2. In what year was Charles de Gaulle elected president of the French Republic for the first time?
3. In what year did Georges Pompidou die?
4. In what year did the Berlin Wall fall?
5. In what year was the law on abortion passed?
6. In what year was the death penalty abolished?
7. When did the first Gulf War (in Iraq) take place?
8. In what year did Hitler come to power?
9. When did Lenin die?
10. In what year was the Vietnam War over?
11. When did the Titanic sink?
12. In what year did Georges Clemenceau die?
13. What is the year of the construction of the Eiffel Tower?
14. In what year did Albertville host the Olympic Games?
15. In what year did Grenoble host the Olympic Games?
16. When did France become the 5^th^ Republic?
17. When was the telephone invented?
18. When did Victor Hugo die?
19. In what year did women in France obtain the right to vote?
20. When did France conduct the last nuclear weapons testing?
21. In what year was the cinema invented?
22. When was the first metro in Paris built?
23. When was the American Navy base Pearl Harbor bombed?
24. In what year was Queen Elizabeth the II crowned?
25. What is the year of the discovery of the rabies vaccine by Pasteur?
26. In what year did Napoleon lose the Battle of Waterloo?
27. When did the United States of America become independent?
28. When did the Soviet Union collapse?
29. In what year was slavery abolished?
30. When was the UNO created?
31. When did Marilyn Monroe die?
32. When did John Lennon die?
33. In what year was Louis XVI of France decapitated?
34. When did Louis XIV of France die?
35. In what year was the Treaty of the European Union signed?
